# Progesterone vs. synthetic progestins and the risk of breast cancer: a systematic review and meta-analysis

**DOI:** 10.1186/s13643-016-0294-5

**Published:** 2016-07-26

**Authors:** Noor Asi, Khaled Mohammed, Qusay Haydour, Michael R. Gionfriddo, Oscar L. Morey Vargas, Larry J. Prokop, Stephanie S. Faubion, Mohammad Hassan Murad

**Affiliations:** 1 Evidence-Based Practice Center, Mayo Clinic Robert D. and Patricia E. Kern Center for the Science of Health Care Delivery, 200 First Street SW, Rochester, MN 55905 USA; 2 Internal Medicine Department, Georgia Regents University, Augusta, USA; 3 Knowledge and Evaluation Research Unit and Mayo Graduate School, Mayo Clinic, Rochester, MN USA; 4 Division of Endocrinology, Diabetes, Metabolism and Nutrition, Mayo Clinic, Rochester, MN USA; 5 Library Public Services, Mayo Clinic, Rochester, MN USA; 6 Division of General Internal Medicine, Women’s Health Clinic, Mayo Clinic, Rochester, MN USA; 7 Division of Preventive, Occupational and Aerospace Medicine, American University of Beirut, 200 First Street SW, Rochester, MN 55905 USA

**Keywords:** Progesterone, Synthetic progestins, Breast cancer, Cardiovascular events, Systematic review, Meta-analysis

## Abstract

**Background:**

Use of menopausal hormonal therapy (MHT)-containing estrogen and a synthetic progestin is associated with an increased risk of breast cancer. It is unclear if progesterone in combination with estrogen carries a lower risk of breast cancer. Limited data suggest differences between progesterone and progestins on cardiovascular risk factors, including cholesterol and glucose metabolism. Whether this translates to differences in cardiovascular outcomes is uncertain. We conducted a systematic review and meta-analysis to synthesize the existing evidence about the effect of progesterone in comparison to synthetic progestins, each in combination with estrogens, on the risk of breast cancer and cardiovascular events.

**Methods:**

We searched MEDLINE, EMBASE, Cochrane Central Register of Controlled Trials, and Scopus through 17 May 2016 for studies that enrolled postmenopausal women using progesterone vs. synthetic progestins and reported the outcomes of interest. Study selection and data extraction were performed by two independent reviewers. Meta-analysis was conducted using the random effects model.

**Results:**

We included two cohort studies and one population-based case-control study out of 3410 citations identified by the search. The included studies enrolled 86,881 postmenopausal women with mean age of 59 years and follow-up range from 3 to 20 years. The overall risk of bias of the included cohort studies in the meta-analysis was moderate. There was no data on cardiovascular events. Progesterone was associated with lower breast cancer risk compared to synthetic progestins when each is given in combination with estrogen, relative risk 0.67; 95 % confidence interval 0.55–0.81.

**Conclusions:**

Observational studies suggest that in menopausal women, estrogen and progesterone use may be associated with lower breast cancer risk compared to synthetic progestin.

**Electronic supplementary material:**

The online version of this article (doi:10.1186/s13643-016-0294-5) contains supplementary material, which is available to authorized users.

## Background

Menopausal hormone therapy (MHT) is highly effective for the treatment of symptoms related to menopause [1]. MHT regimens typically include estrogen and, for women with an intact uterus, a progestin to protect the endometrium from hyperplasia caused by unopposed estrogen. A number of US Food and Drug Administration (FDA)-approved hormone preparations are available for treatment of women with menopausal symptoms [2]. The biochemistry, metabolism, and both beneficial and harmful effects of the various synthetic progestins differ widely from native progesterone and from each other [3].

Micronized progesterone is a bioidentical hormone with a molecular structure identical to that of endogenous progesterone produced by the ovary. Synthetic progestins have a different chemical structure from progesterone. These compounds mimic some of the effects of progesterone but may have different actions on progesterone receptors [4]. Synthetic progestins may be structurally related to progesterone (e.g., medroxyprogesterone acetate (MPA), dydrogesterone) or to testosterone (e.g., levonorgestrel, drospirenone) with differing potency and pharmacokinetics. The physiologic effects of a particular progestin depend not only on these properties but also on receptor binding. In addition to binding to progesterone receptors, these compounds may also have an affinity for androgen, glucocorticoid, and mineralocorticoid receptors [5].

Although some data suggest that MHT increases the risk of breast cancer [6], the risk of breast cancer may differ depending on the type of MHT used. For example, MHT containing conjugated equine estrogens (CEE) and medroxyprogesterone acetate (MPA) has been associated with increased risk of breast cancer compared to CEE alone [7]. Further, breast cancer risk may vary between regimens containing different progestins, with some synthetic progestins exhibiting greater risk than others [8]. The effects of progesterone have been shown to be growth-promoting, neutral, or anti-proliferative in breast cells, whereas in women, synthetic progestins, especially the combination of CEE and MPA, have been found to be growth-promoting [9]. In contrast to progestins, progesterone in combination with estrogen has not been associated with increased breast cancer [8]. Emerging evidence suggests that the progesterone receptor acts as a modulator of estrogen receptor α (ERα) binding and transcription, blocking estrogen-mediated cell proliferation. The presence of progesterone receptors in breast cancer that are positive for ERα is associated with positive clinical outcomes [10].

Progesterone and synthetic progestins also demonstrate varied effects on lipids, coagulation factors, glucose, and insulin and may therefore differentially impact cardiovascular risk, though data are sparse [11].

The PEPI trial previously demonstrated that, when combined with CEE, progesterone, unlike MPA, did not negate the positive effects of CEE on high-density lipoprotein cholesterol (HDL-C) [7]. A recent randomized, double-blind, placebo-controlled trial utilizing 300 mg of progesterone daily showed no adverse changes in endothelial function, blood pressure, weight, or markers of inflammation or coagulation. Although HDL-C was decreased on treatment, the change was not believed to be clinically relevant [12]. We conducted a systematic review and meta-analysis to synthesize the existing evidence about the effect of progesterone compared to synthetic progestins on the risk of breast cancer and cardiovascular disease.

## Methods

A predefined protocol was developed by experts from the Endocrine Society to conduct this systematic review. The protocol included explicit criteria for study selection and plans for the data extraction and analysis. We followed the standards set in the Preferred Reporting Items for Systematic Reviews and Meta-analysis (PRISMA) [13] statement for reporting this review as shown in Additional file 1. This systematic review was submitted for PROSPERO registration but it did not meet registration requirement.

### Eligibility criteria

We included comparative/controlled studies that enrolled women aged 45–59 years who were within 10 years of menopause and received MHT. The studies had to compare estrogen with progesterone (crystalline progesterone preparations) with any of the synthetic progestins in combination with estrogen and report outcomes of interest for a follow-up period ≥6 months. The outcomes of interest were the risk of breast cancer and cardiovascular disease. We excluded non-comparative studies, case series, and non-original papers.

### Literature search

The search included the electronic databases of MEDLINE, MEDLINE in-process and other non-indexed citations, EMBASE, Cochrane Central Register of Controlled Trials and Cochrane Database of Systematic Reviews, and Scopus. We expanded the search to include all languages, with the latest date of inclusion to be 17 May 2016. The database search was conducted by an experienced Mayo Clinic reference librarian. Controlled vocabulary supplemented with keywords was used to search for comparative studies of progesterone vs. synthetic progestins and risk of breast cancer and cardiac events. A manual search for the included studies’ bibliographies and previous relevant systematic review were also conducted. A detailed search strategy is described in the Additional file 2.

### Study selection

Using an online reference management system DistillerSR (Distiller SR, Evidence Partners Incorporated, Ontario, Canada), abstracts and titles that resulted from the electronic search strategy were independently evaluated by two reviewers for potential eligibility, and the full-text versions of all potentially eligible studies were obtained. Two reviewers working independently considered the full-text reports for eligibility. The level of agreement between the two reviewers (*k* level) was 0.7 and 0.8 for abstract screening and full-text screening, respectively. Disagreements were harmonized by consensus and, if not possible, by consensus through arbitration by a third reviewer.

### Data extraction

Using a standardized, piloted, and web-based form (Distiller SR; Evidence Partners Inc.), two reviewers independently extracted data from each study and later reconciled differences, if present. Reviewers determined the methodological quality of studies and collected descriptive, methodological, and outcome data. We extracted the following variables from the studies: study characteristics (study design, inclusion and exclusion criteria), baseline characteristics, and patient demographics, and outcome data.

### Risk of bias assessment

We used a modified Newcastle-Ottawa Scale (NOS) [14] to appraise the risk of bias of the observational studies. The quality of evidence was evaluated using the Grading of Recommendations Assessment, Development and Evaluation (GRADE) [15] methods.

### Data synthesis and statistical analysis

We extracted or calculated the relative risk (RR) of the outcomes of interest with 95 % confidence interval (CI). The *I*^2^ statistic was used to assess heterogeneity of the treatment effect among studies for each outcome. *I*^2^ value >50 % and *p* value <0.10 of the Cochrane Q test suggested substantial heterogeneity that is due to real differences in study populations, protocols, interventions, and/or outcomes. Publication bias was not assessed due to the small number of the studies included. The statistical analyses using DerSimonian and Laird random effects model were performed with CMA version 2 (Biostat, Englewood, New Jersey).

## Results

**Fig. 1:**
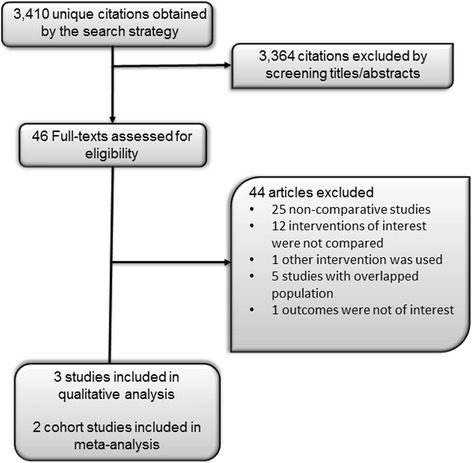
Study selection process. The initial search resulted in 3410 citations. After screening the abstracts, this was limited to 46 potentially relevant articles. These were reviewed in full text by two authors and eventually two cohort studies and one population-based case-control study were included with 44 being excluded

The initial search resulted in 3410 citations. After screening the abstracts, this was limited to 46 potentially relevant articles. These were reviewed in full text by two authors and eventually two cohort studies and one population-based case-control study were included with 44 being excluded for the reasons shown in Fig. 1. The included studies enrolled 86,881 women with a mean age of 59 years and mean follow-up duration of 5 years. None of the studies evaluated the outcome of cardiovascular disease. The studies included are summarized in Table 1. The overall risk of bias of the included cohort studies was moderate. Samples were somewhat representative in the two studies with no baseline imbalance, and the studies were controlled for the most important factors. Table 2 describes the detailed risk of bias assessment of the two included cohort studies.

**Table 1:** Description of included studies

Study	Study population	Age (mean) ±SD	Location	Group 1	Group 2	Type and route of estrogen	Follow-up (mean) in years	Outcomes
Espie et al. [16]	4949 postmenopausal women were included in two groups: exposed group, 2693 postmenopausal women who were receiving MHT or who stopped <5 years, and unexposed group, 2256 postmenopausal women who had never received MHT or who had stopped >5 years. MHT regimes were estradiol alone (351 postmenopausal women), estradiol + natural progesterone and estradiol + synthetic progestins (excluding medroxyprogesterone acetate and 19-nortestosterone derivatives)	60.6 ± 6.3 for exposed group, 64.2 ± 8.3 for unexposed group	France	Estradiol + natural progesterone N1 = 999	Estradiol + synthetic progestins (excluding medroxyprogesterone acetate and 19-nortestosterone derivatives) N2 = 1272	Estradiol transdermal in 78 % and oral in 22 %	2.5	Breast cancer risk
Fournier et al. 2008 [8]	80,377 postmenopausal women were included in two groups: MHT never-users with 23,703 postmenopausal women and MHT ever-users with 56,674 postmenopausal women. MHT regimes were estrogen alone, estrogen + progesterone, estrogen + dydrogesterone, estrogen + other progestins, weak estrogens and other unknown MHT (almost exclusively estradiol compounds)	55.0 ± 4.8 for MHT never-users, 52.3 ± 4.1 for MHT ever-users	France	Estrogen + progesterone (almost exclusively estradiol compounds)	Estrogen + synthetic progestins (almost exclusively estradiol compounds)	Postmenopausal women received either oral or transdermal estrogen (% not reported)	8.1	Breast cancer risk
Cordina-Duverger et al. 2013 [17]	1555 postmenopausal woman, 739 cases treated with combined estrogen and progestagen. 816 controls	Range (25–75)	France	Estrogen + progesterone: 25 cases and 34 controls	Estrogen + synthetic progestins : 55 cases and 43 controls	Not specified	4	Breast cancer risk

*MHT* menopausal hormone therapy

**Table 3:** The effect of progesterone vs. synthetic progestins in combination with estrogen on breast cancer incidence

Study	Group 1	Incidence of breast cancer in group 1	Group 2	Incidence of breast cancer in group 2	Relative risk (RR)	95 % confidence interval (95 % CI)
Espie et al. 2007 [16]	Estradiol + progesterone	4/999	Estradiol + synthetic progestin^a^	12/1272	0.42	0.13–1.31
Fournier et al. 2008 [8]	Estrogen + progesterone (almost exclusively estradiol compounds)	129/40,537 person-years	Estrogen + synthetic progestin (almost exclusively estradiol compounds)	635/135,288 person-years	0.68	0.56–0.82
	Estrogen + progesterone (almost exclusively estradiol compounds)	129/40,537 person-years	Estrogen + synthetic progestin^b^ (almost exclusively estradiol compounds)	606/128,253 person-years	0.67	0.76–0.81

^a^This study excluded users of medroxyprogesterone acetate and 19-nortestosterone derivatives

^b^This is a partial cohort that does not include users of medroxyprogesterone acetate from the analysis

### Meta-analysis

**Fig. 2:**
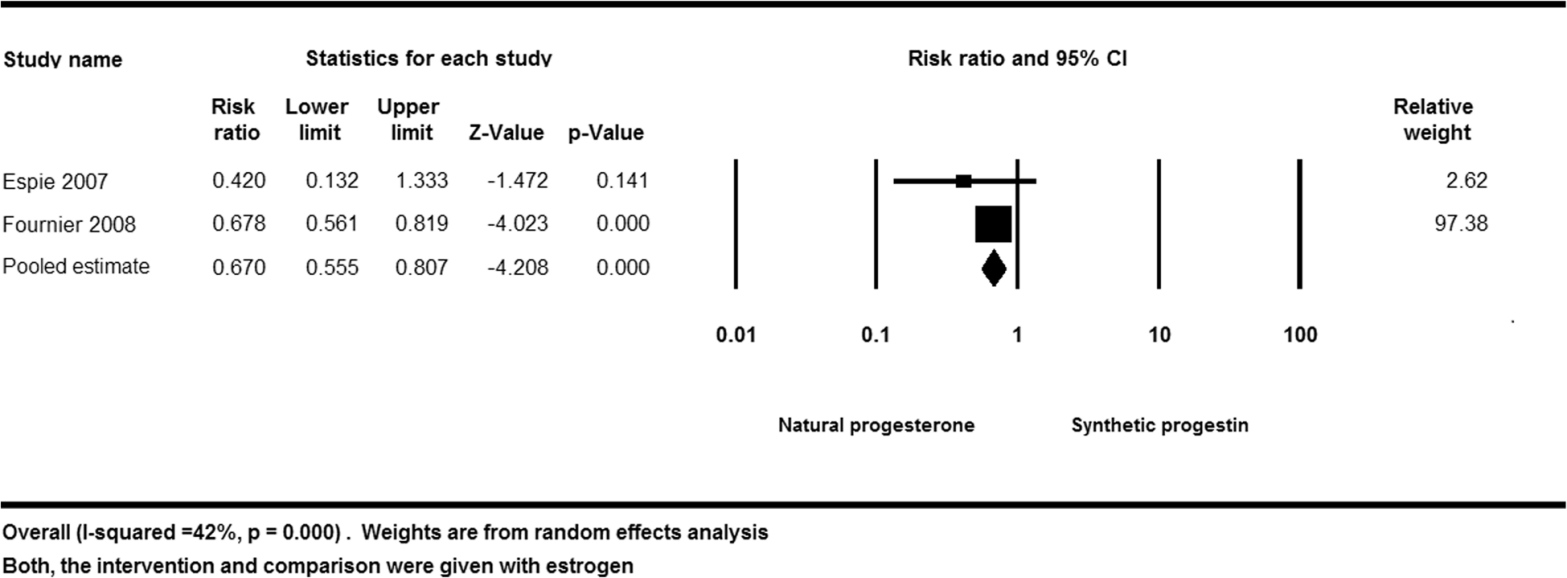
Random effects model of breast cancer risk in postmenopausal women receiving estrogen with progesterone vs. synthetic progestins. This figure shows that progesterone was found to be associated with lower breast cancer compared to synthetic progestins in combination with estrogen

Based on the meta-analysis of the two included cohort studies, progesterone was found to be associated with lower breast cancer risk compared to synthetic progestins in combination with estrogen (RR = 0.67, (95 % CI 0.55–0.81) *I*^2^ = 42 % with *p* value of <0.0001). The quality of evidence was low due to the observational nature of the study design, and Fig. 2 shows the results. Sensitivity analysis was done to exclude postmenopausal women receiving synthetic progestins other than medroxyprogesterone acetate. The number of breast cancer events in women receiving medroxyprogesterone acetate was 29 in 7035 person-years as reported in the study by Fournier et al. [8]. The sensitivity analysis shows no change in results (RR = 0.67 (95 % CI 0.76–0.81) with *p* value of <0.0001).

The effect of combined estradiol and progesterone in comparison with estradiol and synthetic progestin on breast cancer incidence showed a RR 0.42 (95 % CI 0.13–1.31) in Espie [16], whereas Fournier [8] showed a RR 0.68 (95 % CI 0.56–0.82) when comparing postmenopausal women who received estrogen and progesterone to those who received estrogen and synthetic progestin as shown in Table 3.

**Table 4:** The relative risk (95 %) for breast cancer according to the route of administration of estradiol and the type of progestogen used

Study name	Type of MHT	Progesterone	Synthetic progestins
Espie et al. 2007 [16]	Oral estradiol	NR	0.81 (0.23–2.85)
	Transdermal estradiol	0.46 (0.13–1.62)	1.07 (0.50–2.27)

*NR* not reported in the study

The study by Espie [16] reported the route of administration of estradiol where 2101 patients (78 %) received transdermal estradiol and 592 (22 %) received oral estradiol. Subgroup analysis was done based on the route of administration, and no differential effect on the risk of breast cancer was apparent between oral and transdermal routes of administration as shown in Table 4.

**Table 2:** Risk of bias assessment of the included studies

Study	Representativeness of the exposed cohort	Selection of the non-exposed cohort	Ascertainment of exposure	Outcome of interest was not present at start of study	Comparability of cohorts	Assessment of outcome	Adequacy of follow-up cohort
Espie et al. 2007 [16]	Somehow representative	Drawn from the same community	No description	Yes	Study control for most important factors	No description	Yes
Fournier et al. 2008 [8]	Somehow representative	Drawn from the same community	Written self-report questionnaires	Yes	Study control for most important factors	Self-report questionnaires or files of health insurance plan. (Pathology reports were obtained in 96 % of cases)	Yes

We also identified a population-based case-control study that reported similar results. The study showed no significant increased risk of breast cancer among women treated with progesterone in combination with estrogen, odds ratio (OR) 0.80 (CI 95 % 0.44–1.43). However, there was a non-significantly increased risk of breast cancer among users of estrogen with synthetic progestins, OR 1.57 (95 % CI 0.99–2.49) [17].

## Discussion

Based on this systematic review and meta-analysis, progesterone may be associated with lower breast cancer risk compared to synthetic progestins, when each is given in combination with estrogen. No studies have been found reporting on the risk of cardiovascular disease in postmenopausal woman receiving estrogen with progesterone vs. those who are receiving estrogen with synthetic progestins, and no previous systematic reviews have evaluated this question.

Progestin is utilized in MHT regimens for women with an intact uterus to prevent endometrial hyperplasia. Progestins used for MHT regimens can be administered orally, transdermally (patch containing norethisterone/norethindrone), or directly to the endometrium (levonorgestrel intrauterine system). While the levonorgestrel intrauterine system has been shown to be adequate for endometrial protection [18], it is not currently approved by the US FDA for this indication.

The potential role of progestins in increasing breast cancer risk associated with MHT has come under greater scrutiny after the Women’s Health Initiative trial suggested increased risk of breast cancer with continuous use of CEE and MPA for greater than 5 years compared with CEE alone, which showed no increased risk [19]. In fact, CEE alone was associated with a lower risk of breast cancer than placebo after 11 years of observation [20].

Both progesterone and synthetic progestins and the dosing regimen may impact breast cancer risk. In the E3N cohort study, MHT regimens containing estrogen and progesterone or dydrogesterone were not associated with a statistically significant increase in breast cancer risk. All other progestins were associated with an increased risk, with no difference between various progestins [8]. The results of this meta-analysis (which includes the E3N study) are consistent with these findings and show a decreased risk of breast cancer associated with the use of progesterone compared with a progestin (RR = 0.67 (95 % CI 0.76–0.81)).

Progesterone and synthetic progestins may be administered continuously with estrogen or sequentially for 10–14 days per month. Some, but not all, studies comparing these regimens have shown increased risk of breast cancer with continuous dosing compared to sequential dosing [21–23]. Randomized clinical trials are needed to clarify these findings.

### Clinical implications

Accumulating evidence suggests that important differences in risks and benefits exist between various MHT regimens, making individualization of MHT essential. Women with an intact uterus require the use of progesterone for endometrial protection when using systemic estrogen therapy for the management of menopausal symptoms. While an additional study is needed to confirm these results, data suggest lower risk of breast cancer with progesterone and dydrogesterone and do not support a class effect of progestins on breast cancer risk [24]. More studies are needed to define a potential difference in cardiovascular risk between progesterone and synthetic progestins.

### Strengths and limitations

The strength of our review relates to following a predefined protocol, rigorous database search, and duplicate study selection and data extraction. The main limitations are the observational nature of the evidence, which lowers the confidence in the estimates, and the small number of studies included. We were unable to ascertain the presence and impact of publication bias due to the small number of studies. In terms of the individual studies, the major strength of the Fournier study [8] was the inclusion of multiple menopausal hormonal therapies: the regular follow-up implemented in the study. It was the first epidemiological study to provide results indicating that estrogen-progesterone and estrogen-dydrogesterone combinations may be the least harmful MHT in terms of breast cancer risk. However, the results cannot be translated into firm clinical recommendations. The strength of the Espie study [16] was that they studied the previous use of progestins prior to menopause. They found that it had no influence on the risk of breast cancer irrespective of whether or not the women were subsequently exposed to MHT. One limitation of that study was that it was influenced by the common practice in France in which gynecologist avoided prescribing MHT to high-risk women. Lastly, the results of this review are largely influenced by a single large study.

## Conclusions

Observational studies suggest that in menopausal women taking estrogen, progesterone use may be associated with lower breast cancer risk compared to synthetic progestin.

## Abbreviations

MHT, menopausal hormone therapy; CEE, conjugated equine estrogens; MPA, medroxyprogesterone acetate; HDL-C, high-density lipoprotein cholesterol; CI, confidence interval; RR, relative risk; OR, odds ratio; PRISMA, Preferred Reporting Items for Systematic Reviews and Meta-analysis; NOS, Newcastle-Ottawa Scale; GRADE, Grading of Recommendations Assessment, Development and Evaluation

## Additional files

Additional file 1: PRISMA 2009 checklist. We followed the standards set in the Preferred Reporting Items for Systematic Reviews and Meta-analysis (PRISMA) statement for reporting this review. (PDF 84 kb) Additional file 2: Detailed search strategy. We searched MEDLINE, EMBASE, Cochrane Central Register of Controlled Trials, and Scopus through August 2013 (updated search through 17 May 2016 yielded 457 additional abstracts) for studies that enrolled postmenopausal women using progesterone vs. synthetic progestins and reported the outcomes of interest. (PDF 63 kb)
