# Supplementary material for: Progesterone vs. synthetic progestins and the risk of breast cancer: a systematic review and meta-analysis
Source: Syst Rev. 2016 Jul 26;5:121. doi: 10.1186/s13643-016-0294-5 (PMC4960754; doi:10.1186/s13643-016-0294-5)
Supplement: Additional file 2: — Detailed search strategy. We searched MEDLINE, EMBASE, Cochrane Central Register of Controlled Trials, and Scopus through August 2013 (updated search through 17 May 2016 yielded 457 additional abstracts) for studies that enrolled postmenopausal women using progesterone vs. synthetic progestins and reported the outcomes of interest. (PDF 63 kb) [file 13643_2016_294_MOESM2_ESM.pdf]

Supplemental file 1: Detailed Search Strategy

Ovid

Database(s): Embase 1988 to 2013 Week 35, Ovid MEDLINE(R) In-Process & Other Non-Indexed Citations and Ovid MEDLINE(R) 1946 to Present, EBM Reviews - Cochrane Central Register of Controlled Trials July 2013, EBM Reviews - Cochrane Database of Systematic Reviews 2005 to July 2013
Search Strategy:

#	Searches	Results
1	exp Progesterone/	113552
2	exp pregnane derivative/	1699
3	exp algestone/	234
4	exp gestagen/	95689
5	exp progestins/	158811
6	(progesterone* or pregnenedione* or Algestone* or Dihydroprogesterone* or hydroprogesterone* or Hydroxyprogesterone* or Medroxyprogesterone* or alphasone* or alfasone* or methylprogesterone* or methylhydroxyprogesterone* or "adgyn medro*" or didrogesterone or agolutin or akrolutin or colprosterone or corlut* or corluvite or corpomone or "corpus luteum hormone*" or crinone or cyclogest or depoprogesterone* or endometrin or esolut or estima or evapause or flavolutan or fologenon or gepromi or geslutin or gesterol or gestion or gestone or gestormone or gladucorpin or glanducorpin or glanesting or gonadyl or gynlutin or gynolutin or hormoflaveine or hormoluton or "lipo lutin" or "lucorteum sol" or lucorteumsol or lugesteron or luteinique or luteocyclin or luteodyn or luteogan or luteohormone or luteol or luteomersin or luteoris or luteosan or luteostab or luteosterone or luteovis or lutex or lutidon or lutin or lutine or lutocyclin or lutocylin or lutoform or lutogyl or "lutogynestryl fuerte" or lutren or lutromone or macrogestin or mafel or membrettes or nalutron or naturogest or neolutin or "oophormin luteum" or primolut or prochieve or progeffik or progekan or progenin* or progering or progest or progestan or progestasert or progesteroid or progesterol or progesteron or progestin* or progestogel or progestol or progestone or progestronaq or prolidon or proluton or prometrium or prontogest or repogest or silestrus or syngenstrone or syngesterone or ultrogestan or uterogestan or utrogestan or "pregnane derivative*" or norpregnadienes or norpregnanes or norpregnatrienes or norpregnenes or pregnadienediols or pregnadienes or pregnadienetriols or pregnanes or pregnatrienes or pregnenediones or pregnenes or gestagen or gestogen or progestagen* or progestational or progestative or progestogen* or hydroxypregnenolone or dihydroxypregn or allylestrenol or altrenogest or chlormadinone or cyproterone or demegestone or deposiston or desogestrel or dienogest or dimethisterone or drospirenone or dydrogesterone or elcometrine or eltanolone or levonorgestrel or nomegestrol or norethisterone or norgestimate or norgestrel or ethisterone or etonogestrel or "etynodiol diacetate" or	279173

	gestodene or lynestrenol or megestrol or norelgestromin or etynodiol or flugestone or flumedroxone or gestaclone or gestodene or gestonorone or gestrinone or leuprorelin or levonorgestrel or lynestrenol or medrogestone or megestrol or melengestrol or methylestrenolone or nomegestrol or "non ovlon" or norelgestromin or noretynodrel or norgestimate or norgestomet or norgestrienone or osaterone or pregnanediol or pregnanedione or pregnanetriol or prempak or proligestone or promegestone or segesterone or sepranolone or tanaproget or tetrahydroprogesterone or tosagestin or trimegestone or trisequens).mp. [mp=ti, ab, sh, hw, tn, ot, dm, mf, dv, kw, nm, kf, ps, rs, ui, tx, ct]	
7	or/1-6	279921
8	exp *Heart Diseases/	1356038
9	(coronary or heart or myocardi* or cardiac or cardiopath*).ti.	1216945
10	exp *Breast Neoplasms/	389231
11	((breast or mammary or mammaries) adj3 (cancer* or tumor* or tumour* or neoplasm* or carcinoma*)).ti.	319709
12	or/8-11	2282904
13	exp Postmenopause/	63639
14	(postmenopaus* or "post-menopaus*" or ("after" adj3 menopaus*) or ("following" adj3 menopaus*)).mp. [mp=ti, ab, sh, hw, tn, ot, dm, mf, dv, kw, nm, kf, ps, rs, ui, tx, ct]	137660
15	13 or 14	137660
16	7 and 12 and 15	6674
17	exp controlled study/	4218360
18	exp randomized controlled trial/	723013
19	((control$ or randomized) adj2 (study or studies or trial or trials)).mp. [mp=ti, ab, sh, hw, tn, ot, dm, mf, dv, kw, nm, kf, ps, rs, ui, tx, ct]	5464633
20	meta analysis/	125634
21	meta-analys$.mp.	203300
22	exp "systematic review"/	63637
23	(systematic* adj review$).mp.	156216
24	exp Cohort Studies/	1605366
25	exp longitudinal study/	1026986
26	exp retrospective study/	816646
27	exp prospective study/	670721
28	exp comparative study/	2492723
29	exp clinical trial/	1728244
30	exp cross-sectional study/	283515
31	crossover procedure/	38209
32	exp cross-over studies/	97444
33	multivariate analysis/	183723

34	((clinical or comparative or cohort or longitudinal or retrospective or prospective or concurrent or "cross- sectional" or crossover or "cross-over") adj (study or studies or survey or surveys or analysis or analyses or trial or trials)).mp.	7178988
35	("crossover procedure" or "cross-over procedure" or "multivariate analys*").mp. [mp=ti, ab, sh, hw, tn, ot, dm, mf, dv, kw, nm, kf, ps, rs, ui, tx, ct]	375337
36	("case control study" or "case control studies").mp.	322722
37	exp case control study/	751329
38	exp Randomized Controlled Trials as Topic/	146103
39	or/17-38	11093295
40	16 and 39	4832
41	from 16 keep 3682-6139	2458
42	limit 41 to (clinical trial, all or clinical trial, phase i or clinical trial, phase ii or clinical trial, phase iii or clinical trial, phase iv or clinical trial or comparative study or controlled clinical trial or evaluation studies or guideline or meta analysis or multicenter study or practice guideline or randomized controlled trial or systematic reviews) [Limit not valid in Embase,CCTR,CDSR; records were retained]	855
43	40 or 42	4851
44	limit 43 to (book or book series or editorial or erratum or letter or note or addresses or autobiography or bibliography or biography or comment or dictionary or directory or interactive tutorial or interview or lectures or legal cases or legislation or news or newspaper article or overall or patient education handout or periodical index or portraits or published erratum or video-audio media or webcasts) [Limit not valid in Embase,Ovid MEDLINE(R),Ovid MEDLINE(R) In-Process,CCTR,CDSR; records were retained]	229
45	43 not 44	4622
46	from 16 keep 6140-6674	535
47	45 or 46	4791
48	animals/ not humans/	4637755
49	47 not 48	4776
50	limit 49 to female [Limit not valid in CCTR,CDSR; records were retained]	4107
51	49 and (female* or women or woman).mp. [mp=ti, ot, ab, sh, hw, kw, tn, dm, mf, dv, nm, kf, ps, rs, an, ui, tx, ct]	4501
52	50 or 51	4544
53	remove duplicates from 52	2921

Scopus

1 TITLE-ABS-KEY(progesterone* or pregnenedione* or Algestone* or Dihydroprogesterone* or hydroprogesterone* or Hydroxyprogesterone* or Medroxyprogesterone* or alphasone* or alfasone* or methylprogesterone* or methylhydroxyprogesterone* or "adgyn medro*" or didrogesterone or agolutin or akrolutin or colprosterone or corlut* or corluvite or corpomone or "corpus luteum hormone*" or crinone or cyclogest or depoprogesterone* or endometrin or esolut or estima or evapause or flavolutan or fologenon or gepromi or geslutin or gesterol or gestion or gestone or gestormone or gladucorpin or glanducorpin or glanesting or gonadyl or gynlutin or gynolutin or hormoflaveine or hormoluton or "lipo lutin" or "lucorteum sol" or lucorteumsol or lugesteron or luteinique or luteocyclin or luteodyn or luteogan or luteohormone or luteol or luteomersin or luteoris or luteosan or luteostab or luteosterone or luteovis or lutex or lutidon or lutin or lutine or lutocyclin or lutocylin or lutoform or lutogyl or "lutogynestryl fuerte" or lutren or lutromone or macrogestin or mafel or membrettes or nalutron or naturogest or neolutin or "oophormin luteum" or primolut or prochieve or progeffik or progekan or progenin* or progering or progest or progestan or progestasert or progesteroid or progesterol or progesteron or progestin* or progestogel or progestol or progestone or progestronaq or prolidon or proluton or prometrium or prontogest or repogest or silestrus or syngenstrone or syngesterone or ultrogestan or uterogestan or utrogestan or "pregnane derivative*" or norpregnadienes or norpregnanes or norpregnatrienes or norpregnenes or pregnadienediols or pregnadienes or pregnadienetriols or pregnanes or pregnatrienes or pregnenediones or pregnenes or gestagen or gestogen or progestagen* or progestational or progestative or progestogen* or hydroxypregnenolone or dihydroxypregn or allylestrenol or altrenogest or chlormadinone or cyproterone or demegestone or deposiston or desogestrel or dienogest or dimethisterone or drospirenone or dydrogesterone or elcometrine or eltanolone or levonorgestrel or nomegestrol or norethisterone or norgestimate or norgestrel or ethisterone or etonogestrel or "etynodiol diacetate" or gestodene or lynestrenol or megestrol or norelgestromin or etynodiol or flugestone or flumedroxone or gestaclone or gestodene or gestonorone or gestrinone or leuprorelin or levonorgestrel or lynestrenol or medrogestone or megestrol or melengestrol or methylestrenolone or nomegestrol or "non ovlon" or norelgestromin or noretynodrel or norgestimate or norgestomet or norgestrienone or osaterone or pregnanediol or pregnanedione or pregnanetriol or prempak or proligestone or promegestone or segesterone or sepranolone or tanaproget or tetrahydroprogesterone or tosagestin or trimegestone or trisequens)

2 TITLE(coronary or heart or myocardi* or cardiac or cardiopath* or (breast W/3 cancer*) or (breast W/3 tumor*) or (breast W/3 tumour*) or (breast W/3 neoplasm*) or (breast W/3 carcinoma*) or (mammary W/3 cancer*) or (mammary W/3 tumor*) or (mammary W/3 tumour*) or (mammary W/3 neoplasm*) or (mammary W/3 carcinoma*) or (mammaries W/3 cancer*) or (mammaries W/3 tumor*) or (mammaries W/3 tumour*) or (mammaries W/3 neoplasm*) or (mammaries W/3 carcinoma*))

3 TITLE-ABS-KEY(postmenopaus* or "post-menopaus*" or ("after" W/3 menopaus*) or ("following" W/3 menopaus*))

4 TITLE-ABS-KEY((meta W/1 analys*) OR (systematic* W/2 review*) OR (control* W/2 stud*) OR (control* W/2 trial*) OR (randomized W/2 stud*) OR (randomized W/2 trial*) or "comparative stud*" OR "comparative survey*" OR "comparative analys*" OR "cohort stud*" OR "cohort survey*" OR "cohort analys*" OR "longitudinal stud*" OR "longitudinal survey*" OR "longitudinal analys*" OR "retrospective stud*" OR "retrospective survey*" OR "retrospective analys*" or "prospective stud*" OR "prospective survey*" OR "prospective analys*" or "concurrent stud*" OR "concurrent survey*" OR "concurrent analys*" or "clinical stud*" OR "clinical trial*" or "cross-sectional stud*" or "cross-sectional analys*" or "cross-over stud*" or "cross-over analys*" or "cross-over procedure" or "crossover stud*" or "crossover analys*" or "crossover procedure" or "multivariate analys*" or "case control study" or "case control studies")

5 1 and 2 and 3 and 4

6 PMID(0*) OR PMID(1*) OR PMID(2*) OR PMID(3*) OR PMID(4*) OR PMID(5*) OR PMID(6*) OR PMID(7*) OR PMID(8*) OR PMID(9*)

7 5 and not 6

8 DOCTYPE(le) OR DOCTYPE(ed) OR DOCTYPE(bk) OR DOCTYPE(er) OR DOCTYPE(no) OR DOCTYPE(sh)

9 7 and not 8
